# Efficacy of Computer-Aided Three-Dimensional Ultrasound Guidance for Neuraxial Anesthesia in Adult Patients: A Systematic Review and Meta-Analysis

**DOI:** 10.7759/cureus.72657

**Published:** 2024-10-29

**Authors:** Yuji Kamimura, Hidekazu Ito, Tatsuya Tsuji, Toshiyuki Nakanishi, Kazuya Sobue

**Affiliations:** 1 Department of Anesthesiology and Intensive Care Medicine, Nagoya City University Graduate School of Medical Sciences, Nagoya, JPN; 2 Department of Anesthesiology, Toyokawa City Hospital, Toyokawa, JPN; 3 Department of Anesthesiology, Okazaki City Hospital, Okazaki, JPN

**Keywords:** artificial intelligence, epidural anesthesia, lumbar puncture, spinal anesthesia, systematic review, ultrasound

## Abstract

Artificial intelligence for ultrasound scanning in regional anesthesia is a rapidly developing interdisciplinary field. This study aimed to evaluate the efficacy of computer-aided three-dimensional ultrasound (C-aided US) guidance for neuraxial anesthesia in adult patients.

We searched all randomized controlled trials (RCTs) of adult patients who required neuraxial anesthesia in the MEDLINE, CENTRAL, Embase, International Clinical Trials Registry Platform (ICTRP), and ClinicalTrials.gov databases on June 19, 2023. The primary outcomes were first-pass success, procedure time, and incidence of procedure-related adverse events. We used the Risk of Bias 2 to evaluate the risk of bias for each outcome, a random-effects model to conduct a meta-analysis, and the Grading of Recommendations Assessment, Development, and Evaluation (GRADE) approach to evaluate the certainty of evidence.

Seven RCTs (594 patients) were included. The C-aided US guidance results were as follows: first-pass success (risk ratio = 1.39, 95% confidence interval (CI) = 0.89 to 2.16; low certainty) and total procedure time (mean difference = 0.85 minutes, 95% CI = −0.81 to 2.5; low certainty). Four RCTs reported procedure-related adverse events (e.g., paresthesia, back pain, hemorrhagic events) in both groups (low certainty).

The updated meta-analysis showed that there might be no differences in the first-pass success rates and total procedure times between C-aided US guidance and anatomical landmark guidance for neuraxial punctures.

## Introduction and background

Artificial intelligence (AI) for ultrasound scanning in regional anesthesia is a rapidly developing interdisciplinary field [1]. Recently, computer-aided three-dimensional ultrasound (C-aided US) has been developed for neuraxial anesthesia. This new portable ultrasound device uses AI-driven detection algorithms to automatically identify intervertebral space on ultrasound images and point of needle insertion on the skin [1]. Such advances not only streamline the procedure but also have the potential to improve patient outcomes during neuraxial anesthesia.

Several randomized controlled trials (RCTs) have compared the efficacy of C-aided US guidance with the conventional anatomical landmarks procedures for neuraxial anesthesia, suggesting that C-aided US can lead to better outcomes [2,3]. Moreover, previous systematic reviews and meta-analyses have demonstrated that the use of C-aided US for neuraxial procedures increased the first-pass success rate [4,5]. However, the small sample sizes of these studies have limited their generalizability, remaining a topic of ongoing discussion and requiring further investigation.

We performed an updated systematic review and meta-analysis to rigorously evaluate the efficacy of C-aided US guidance for neuraxial anesthesia in adult patients. By synthesizing the most recent evidence, our objective is to provide more definitive insights into the clinical benefits and potential limitations of this innovative approach.

## Review

Methodology

We followed the Preferred Reporting Items for Systematic Reviews and Meta-Analyses (PRISMA) 2020 guidelines [6] and adhered to the recommendations outlined in the Cochrane Handbook [7]. We registered our protocol in the Open Science Framework (https://osf.io/az8bv/).

Inclusion and Exclusion Criteria

This study aimed to assess the efficacy of C-aided US guidance for neuraxial anesthesia in adult patients compared with that of anatomical landmarks. The study included all adult participants (≥18 years) who required neuraxial anesthesia (spinal anesthesia, epidural anesthesia, and combined spinal-epidural anesthesia) performed with C-aided US guidance or landmark guidance. The intervention group used C-aided US guidance (Accuro; Rivanna, Charlottesville, VA, USA) for neuraxial anesthesia. The control group used traditional anatomical landmark guidance for neuraxial anesthesia.

We included RCTs that assessed the efficacy of C-aided US guidance for neuraxial anesthesia in adult patients. We did not apply country restrictions or language and examined every paper, including articles, letters, and conference abstracts. We did not exclude studies based on the publication year or observation period. We excluded crossover trials, quasi-randomized trials, and quasi-experimental studies.

Outcomes

The primary outcomes were the first-pass success rate, procedure time, and adverse events. We defined first-pass success as when the needle successfully achieved the target space in a single attempt without redirection [8]. The procedure time (preparation time plus insertion time) and adverse events were defined by the authors of the study. We summarized the incidence number and proportion of all adverse events. The numerator was the number of participants experiencing adverse reactions, and the denominator was the total number of participants.

The secondary outcomes were the first-attempt success and patient satisfaction. We defined first-attempt success as a successful single skin puncture with the needle [8,9]. We did not count redirection in the skin. We defined patient satisfaction as that determined by the original authors and applied no restrictions regarding the scale (e.g., four-point scale (1 intolerable; 2 painful; 3 unpleasant; 4 comfortable), five-point scale (1 very unpleasant; 2 unpleasant; 3 satisfactory; 4 good; 5 very good)). We converted the patient satisfaction scores reported in the original studies to a five-point scale.

Search Strategy

We searched the following databases on June 19, 2023: Medline (PubMed), Cochrane Central Register of Controlled Trials (Cochrane Library), and Embase (Dialogue). We also searched the following databases for ongoing or unpublished trials: the World Health Organization International Clinical Trials Registry Platform (ICTRP) search portal and ClinicalTrials.gov. The search strategies are available at https://osf.io/az8bv/. We reviewed the reference lists of included studies, international guidelines, as well as articles citing the eligible studies and their reference lists.

Selection of the Process

After removing duplicates, two independent reviewers (YK and HI) screened the titles and abstracts, followed by a full-text assessment to determine eligibility. When relevant data were missing, we contacted the original authors. Any disagreements between the two reviewers were resolved through discussion, and if consensus could not be reached, a third reviewer (TT) acted as an arbiter.

Data Extraction

Two independent reviewers (YK and HI) used a standardized data collection form to perform independent data extraction of the included studies [10]. We collected the study background (author, year, country, number of patients, indication, neuraxial technique, patient’s position, difficulty, experience of the sonographer, and experience of the interventionist) and outcomes (first-pass success, procedure time, adverse events, first-attempt success, and patient satisfaction). Any disagreements were resolved by discussion, and if this failed, a third reviewer acted as an arbiter (TT).

Quality Assessment

Two independent reviewers (YK and HI) used the Risk of Bias 2 tool to independently evaluate the risk of bias [11]. Disagreements between the two reviewers were discussed, and if this failed, a third reviewer (TT) acted as an arbiter.

Measures of Treatment Effects

We pooled the risk ratios (RRs) and 95% confidence intervals (CIs) for first-pass success and first-attempt success, the mean differences (MDs) and 95% CIs for procedure time, and the standardized mean differences (SMDs) and 95% CIs for patient satisfaction. We summarized adverse events according to the definition in the original article, but we did not perform a meta-analysis.

Synthesis Methods

Missing data: As much as possible, we performed an intention-to-treat analysis for all data. We asked for data not presented by the original authors. For continuous data, we did not impute missing data, adhering to the guidance provided by the Cochrane Handbook [7]. We conducted a meta-analysis using the available data from the original studies. In cases where the studies only reported standard errors or p-values, we employed Altman’s method to derive standard deviations [12]. If these values could not be obtained after contacting the authors, standard deviations were estimated from CIs and t-values based on recommendations from the Cochrane Handbook [7] or other validated methods [12]. The validity of these calculations was evaluated through sensitivity analysis.

Heterogeneity: Statistical heterogeneity was evaluated through visual inspection of forest plots and by calculating the I² statistic. In cases where significant heterogeneity was identified (I² > 50%), potential sources were explored. The Cochrane chi-square test was employed for the I² calculation, with p-values below 0.10 considered indicative of statistical significance.

Meta-Analysis

We used Review Manager software (Rev Man 5.4.2) to perform a meta-analysis with a random-effects model.

Sensitivity Analysis

We conducted sensitivity analyses on the primary outcomes to assess whether the review results were robust enough to justify the decisions made throughout the review process. Studies with high or some concern in the overall assessment of the Risk of Bias 2 were excluded [11].

Reporting Bias

We searched the clinical trial registry systems (ClinicalTrials.gov and ICTRP) and performed an extensive literature search for unpublished trials. To evaluate outcome reporting bias, we compared the outcomes specified in the trial protocols with those reported in the publications.

Certainty Assessment

Two reviewers (YK and TT) evaluated the certainty of the evidence using the Grading of Recommendations, Assessment, Development, and Evaluation (GRADE) framework [13]. Any disagreements between the two were resolved through discussion, and if a consensus could not be reached, a third reviewer (HI) served as an arbiter. A summary of findings table was created based on the guidelines in the Cochrane Handbook [7] for the following outcomes: first-pass success, procedure time, adverse events, first-attempt success, and patient satisfaction.

Differences Between the Study Protocol and the Review

We were unable to conduct a subgroup analysis due to the limited availability of data. Second, we could not undertake the following sensitivity analyses: (1) exclusion of studies using imputed statistics because of no studies, and (2) only the participants who completed the study with complete data because of no studies.

Results

Search Results and Characteristics of the Included Trials

**Figure 1:**
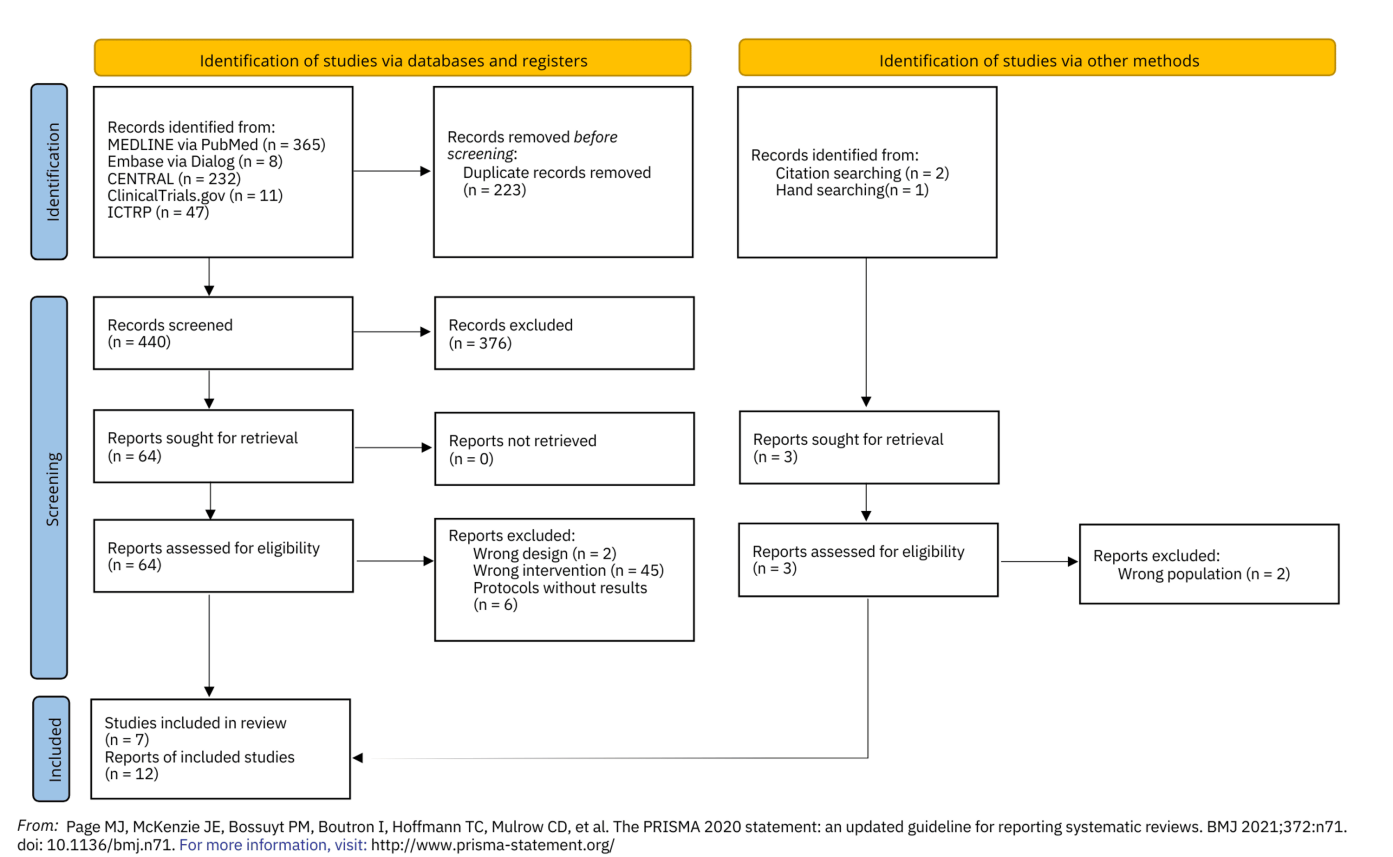
Preferred Reporting Items for Systematic Reviews and Meta-Analyses (PRISMA) 2020 flow diagram.

Following the removal of duplicates, 440 records were identified during a search conducted on June 19, 2023. After a full-text screening for eligibility, 64 reports were reviewed. Of these, 53 reports were excluded, and seven studies involving a total of 594 patients fulfilled all of the eligibility criteria [2,3,14-18] (Figure 1) (Supplementary Table: https://osf.io/az8bv/]).

The characteristics of the included studies are presented in Table 1. Obstetric anesthesia, such as cesarean delivery and labor analgesia, was the indication for the neuraxial procedure in five studies [2,3,14,17,18]. The remaining two studies were lower limb orthopedic surgery [15] and surgical anesthesia patients [16]. Neuraxial techniques varied, with three studies involving spinal puncture [14,15,18], two studies using combined spinal-epidural techniques [2,3], and one study involving epidural puncture [16]. One study [17] involved either a spinal puncture or a combined spinal-epidural puncture. In three studies [2,15,18], the neuraxial technique was predicted to be a difficult setting, whereas it was predicted to be a heterogeneous setting in four studies [3,14,16,17]. Further, in three studies [2,3,18], neuraxial US was conducted by an experienced sonographer.

**Table 1:** Characteristics of the included studies. C: control; CSE: combined spinal–epidural; I: intervention; N: total number

Reference	Country	Number of patients, N (I/C)	Indication	Neuraxial technique	Position	Predicted difficulty	Experience of the sonographer	Experience of the interventionist
Bae et al. (2023) [3]	Korea	84 (42/42)	Labor analgesia	CSE	Lateral	Heterogeneous	The three anesthesiologists had performed over 50 neuraxial ultrasound scans before this study	The CSE procedures were performed by one of the three anesthesiologists
Delforche et al. (2021)[15]	Belgium	58 (29/29)	Cesarean delivery	Spinal	Sitting	Heterogeneous	Unclear	Experience varied, but residents were expected to have performed at least 10 spinal punctures before. A resident initiated 24%
Ghisi et al. (2019) [16]	Italy	130 (65/65)	Lower limb orthopedic surgery	Spinal	Sitting or lateral	Body mass index ≥30 kg/m^2^	Unclear	Each spinal block was performed by anesthesiologists skilled in both techniques with a 25- or 27-gauge needle
Kimizuka et al. (2022) [17]	Japan	60 (30/30)	Surgical anesthesia patients	Epidural	Lateral	Heterogeneous	A novice resident (resident A) or senior residents (resident B, C)	A novice resident (resident A) or by senior residents (resident B, C)
Ni et al. (2021) [2]	China	80 (40/40)	Cesarean delivery	CSE	Sitting	Body mass index >30 kg/m^2^	A single anesthesiologist was very familiar with Accuro after systematic learning before starting this study	Experienced anesthesiologists with >5 years of clinical experience in CSE
Singla et al. (2019) [18]	USA	142 (47/95)	Cesarean delivery	Spinal or CSE	Sitting	Heterogeneous	All residents watched a 10-minute video and received 20 minutes of hands-on training before participating in the study	Anesthesiology residents
Weiniger et al. (2022) [19]	Israel	40 (18/22)	Cesarean delivery	Spinal	Unclear	Body mass index >30 kg/m^2^ and impalpable bony landmarks	Trained anesthesiologists	The duty anesthesiologist, resident, or senior physician (<2 years, ≥2 to <5 years, ≥5 years of experience)

**Figure 2:**
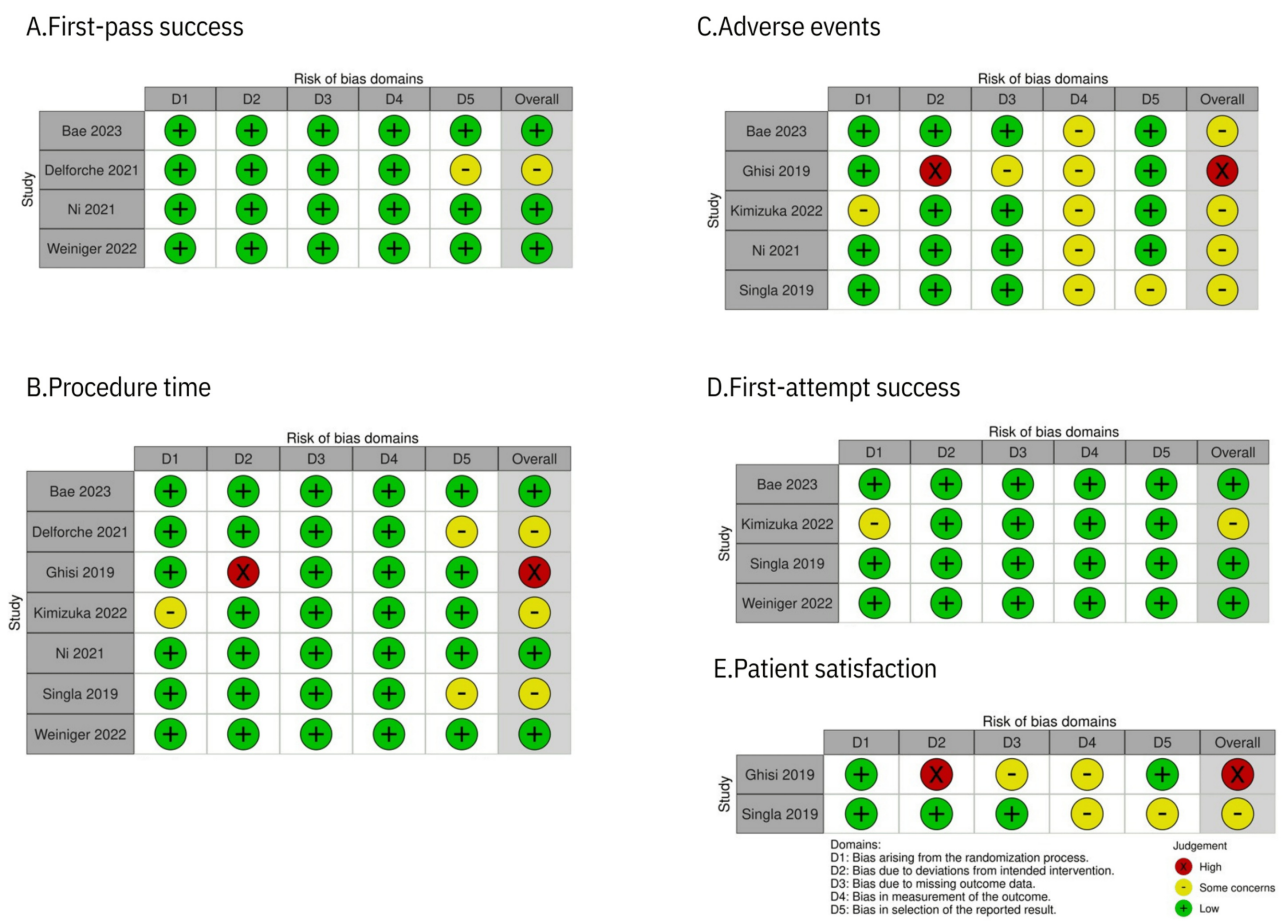
Risk of bias assessment. Bae et al. (2023) [3]; Delforche et al. (2021) [14]; Ni et al. (2021) [2]; Weiniger et al. (2022) [18]; Ghisi et al. (2019) [15]; Kimizuka et al. (2022) [16]; Singla et al. (2019) [17].

Figure 2 presents a summary of the risk of bias for each outcome in the included studies, with ratings ranging from low to high. For first-pass success, 75% of the overall risk of bias was low risk, and 25% was some concerns. Regarding procedure time, 43% of the overall risk of bias was low risk, 43% was some concerns, and 14% was high risk. Concerning adverse events, 80% of the overall risk of bias was some concerns, and 20% was high risk.

Primary Outcomes

Table 2 provides the summary of the findings of this study (for more detailed information, refer to Supplementary Table: https://osf.io/az8bv/).

**Table 2:** Summary of findings. ^a^: Downgraded by one level for imprecision (small sample size, n = 262). ^b^: Downgraded by one level, as there are two unpublished and one ongoing protocol with first-pass success as the primary outcome. ^c^: Downgraded by one level for inconsistency (I² = 89%, p-value for heterogeneity <0.05). ^d^: Downgraded by one level for imprecision (95% CI includes no clinical effect and a clinical effect). ^e^: Periprocedural composite complications were defined as radicular pain, paresthesia, bloody tap, and an accidental dural puncture with an epidural needle during the combined spinal–epidural procedure. ^f^: Downgraded by one level for risk of bias (four of five studies were rated as some concerns and the remaining one had a high risk of bias). ^g^: Downgraded by one level, as there are unpublished and ongoing protocols with adverse events. ^h^: Downgraded by one level for imprecision (small sample size, n = 326; and the 95% CI includes no clinical effect and a clinical effect). ^i^: Downgraded by one level, as there are two unpublished and one ongoing protocol with first attempt success as the primary outcome. ^j^: Downgraded by one level for risk of bias (one of two studies was at a high risk of bias and the remaining one had some concerns). ^k^: Downgraded by one level for imprecision (small sample size, n = 241). *The risk in the intervention group (and its 95% CI) is based on the assumed risk in the comparison group and the relative effect of the intervention (and its 95% CI). CI: confidence interval; MD: mean difference; RR: risk ratio; SMD: standardized mean difference; RCTs: randomized controlled trials

Summary of findings: Efficacy of computer-aided three-dimensional ultrasound guidance for neuraxial anesthesia in adult patients						
Computer-aided three-dimensional neuraxial ultrasound guidance compared with anatomical landmark guidance for neuraxial procedures						
Patient or population: All adult participants (≥18 years) who require neuraxial punctures (spinal, epidural, combined spinal-epidural anesthesia, and lumbar puncture). Setting: Anesthesia during surgery and labor, and other diagnostic procedures. Intervention: computer-aided three-dimensional neuraxial ultrasound guidance. Comparison: Anatomical landmarks						
Outcomes	Anticipated absolute effects^*^ (95% CI)		Relative effect (95% CI)	Number of participants (studies)	Certainty of the evidence (GRADE)	Comments
	Risk with landmark	Risk with ultrasound				
First-pass success	331 per 1,000	460 per 1,000 (294 to 715)	RR = 1.39 (0.89 to 2.16)	262 (4 randomized controlled trials (RCTs))	⨁⨁◯◯ Low^a,b^	
Procedure time	The mean procedure time ranged from 2 to 15.5 minutes	Mean difference = 0.85 minute higher (0.81 lower to 2.51 higher)	-	563 (7 RCTs)	⨁⨁◯◯ Low^c,d^	
Adverse events	One study reported one and three cases of paresthesia and one and zero cases of bloody tap in the intervention and control groups, respectively. One study reported three and 18 paresthesia and three and six cases of blood in epidural catheter in the intervention and control groups, respectively. One study reported three and two cases of back pain and zero and two dural puncture cases in the intervention and control groups, respectively. One study reported six and nine perioperative composite complications in the intervention and control groups. The remaining one study reported no adverse events^e^			465 (5 RCTs)	⨁⨁◯◯ Low^f,g^	
First-attempt success	482 per 1,000	656 per 1,000 (521 to 820)	RR = 1.36 (1.08 to 1.70)	326 (4 RCTs)	⨁⨁◯◯ Low^h,i^	
Patient satisfaction	-	SMD = 0 SD (0.26 lower to 0.26 higher)	-	241 (2 RCTs)	⨁⨁◯◯ Low^j,k^	
GRADE Working Group grades of evidence: High certainty: the true effect lies close to that of the estimate of the effect. Moderate certainty: the true effect is likely to be close to the estimate of the effect, but there is a possibility that it is substantially different. Low certainty: the true effect may be substantially different from the estimate of the effect. Very low certainty: the true effect is likely to be substantially different from the estimate of effect						

First-Pass Success

**Figure 3:**
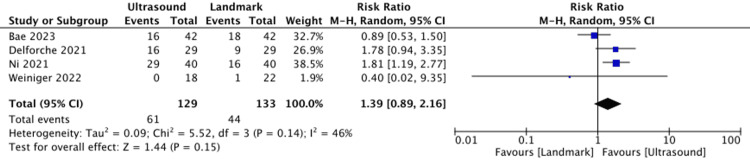
Forest plot for first-pass success. Bae et al. (2023) [3]; Delforche et al. (2021) [14]; Ni et al. (2021) [2]; Weiniger et al. (2022) [18]. CI: confidence interval

Compared with anatomical landmark guidance, C-aided US guidance for neuraxial punctures might not increase first-pass success (four studies, 262 participants: RR = 1.39, 95% CI = 0.89 to 2.16, I^2^ = 46%; low certainty of evidence) (Figure 3). We conducted a sensitivity analysis of studies with a low overall Risk of Bias 2 assessment; however, the results were similar to those obtained in the original analysis (three studies, 204 participants: RR = 1.23, 95% CI = 0.65 to 2.36, I^2^ = 60%) (Supplementary Figure: https://osf.io/az8bv/).

Procedure Time

**Figure 4:**
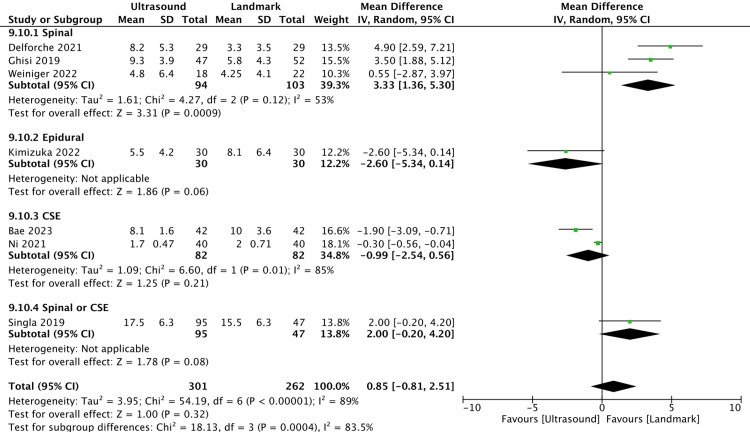
Forest plot for procedure time. Delforche et al. (2021) [14]; Ghisi et al. (2019) [15]; Weiniger et al. (2022) [18]; Kimizuka et al. (2022) [16]; Bae et al. (2023) [3]; Ni et al. (2021) [2]; Singla et al. (2019) [17]. CI: confidence interval; CSE: combined spinal-epidural anesthesia

Compared with anatomical landmark guidance, C-aided US guidance for neuraxial punctures might not reduce procedure time (seven studies, 563 participants: MD = 0.85 minutes, 95% CI = −0.81 to 2.51, I^2^ = 89%; low certainty of evidence) (Figure 4). We conducted a sensitivity analysis of studies with a low overall Risk of Bias 2 assessment; however, the results were consistent with those found in the original analysis (three studies, 204 participants: MD = −0.80 minutes, 95% CI = −2.12 to 0.52, I^2^ = 71%) (Supplementary Figure: https://osf.io/az8bv/).

Adverse Events

Five studies (465 participants) assessed adverse events. One study [15] reported one and three paresthesias, respectively, and one and zero bloody taps, respectively, in the intervention and control groups. One study [2] reported three and 18 paresthesias, respectively, and three and six cases of blood in epidural catheters, respectively, in the intervention and control groups. One study [17] reported three and two back pain cases, respectively, and zero and two dural punctures, respectively, in the intervention and control groups. One study [3] reported six and nine perioperative composite complications, respectively, in the intervention and control groups. The remaining study [16] reported no adverse events.

Secondary Outcomes

Compared with anatomical landmark guidance, the C-aided US guidance for neuraxial punctures might increase first-attempt success (four studies, 326 participants: RR = 1.36, 95% CI = 1.08 to 1.70, I^2^ = 22%; low certainty of evidence) (Supplementary Figure: https://osf.io/az8bv/). We found that C-aided US guidance for neuraxial punctures might not increase patient satisfaction (two studies, 241 participants: SMD = 0.0, 95% CI = −0.26 to 0.26, I^2^ = 0%; low certainty of evidence) (Supplementary Figure: https://osf.io/az8bv/).

The PRISMA 2020 checklist of the present study is available in the Open Science Framework (https://osf.io/az8bv/).

Discussion

We compared the efficacy of C-aided US guidance with anatomical landmark guidance for neuraxial anesthesia in adult patients and found that there might be no difference in the first-pass success and total procedure time between the two types of guidance.

These results differ from those of a previous systematic review and meta-analysis showing that C-aided US guidance for neuraxial punctures had a favorable risk ratio for first-pass success (RR = 1.44; 95% CI = 1.01 to 2.05, p = 0.05) [4]. However, a previous review [4] contained some data extracted from the first-attempt success. The first-attempt success was defined as a successful single skin puncture with the needle, not to count redirection in the skin. The first-pass success was defined as when the needle successfully achieved the target space in a single attempt without redirection [8]. Hence, it did not evaluate first-pass success correctly. Therefore, we think that our findings showed no difference in the first-pass success between the two types of guidance providing a more reliable result.

We found that there might be no difference in the procedure time between C-aided US guidance and anatomical landmark guidance, even with an increased first-attempt success rate when using C-aided US guidance for neuraxial punctures. A previous systematic review and meta-analysis of conventional pre-procedural US guidance in obstetrics found that there was no difference in the total time between pre-procedural US and landmark palpation, with an MD of 50.1 seconds (95% CI = 13.7 to 113.9, p = 0.129) in eight trials with 709 patients [19]. US guidance may shorten the insertion time, but the total time remained the same when adding the preparation time. C-aided US guidance was also considered to be consistent with this trend. In our study, the subgroup analysis focusing on spinal anesthesia (three studies, 197 participants: MD = 3.33 minutes, 95% CI = 1.36 to 5.30, I² = 53%) revealed a significantly faster anatomical landmark procedure. This represents a novel finding and suggests that differences between techniques may exist. However, further studies are required to confirm these differences and establish a clearer understanding of the implications across procedures.

Regarding adverse events, such as paresthesia, fewer adverse events might be observed with C-aided US guidance than with anatomical landmark guidance for neuraxial punctures. A previous systematic review and meta-analysis of conventional pre-procedural US guidance showed a 73% reduction in the risk of a traumatic procedure [20]. The use of C-aided US can potentially reduce the complications of neuraxial anesthesia. However, we only summarized the results; therefore, these results are insufficient to suggest the safety of C-aided US guidance.

The strengths of our study included conducting a comprehensive search in accordance with the PRISMA statement [6] and evaluating the certainty of evidence using the GRADE approach [13]. However, our study has several limitations. First, the included studies varied in study designs, patient populations, and outcome measures, which may have introduced heterogeneity. Second, some assessments of the overall risk of bias were rated as high or some concerns. To mitigate this, we conducted a sensitivity analysis excluding studies with high or some concerns in the Risk of Bias 2 assessment [11] and confirmed that the results remained consistent. Finally, we included only seven studies, which represents a relatively small sample size. Therefore, further high-quality RCTs are needed to confirm the efficacy and safety of C-aided US guidance for neuraxial anesthesia in adult patients. Furthermore, conducting a trial sequential analysis would be warranted to strengthen the conclusions, particularly when analyzing outcomes across different neuraxial techniques. The C-aided US guidance may, in the future, prove to be superior, particularly with advancements in the psychomotor domain, if incorporated into teaching methodologies.

## Conclusions

This updated review suggests that there might be no difference in the first-pass success and total procedure time between C-aided US guidance and anatomical landmark guidance for neuraxial punctures. Regarding adverse events, fewer adverse events might be observed with C-aided US guidance than with anatomical landmark guidance for neuraxial punctures, although our analysis only summarized these results. Given the continuous advancements in US technology and its integration into clinical practice, further high-quality RCTs with larger sample sizes are necessary to draw more definitive conclusions.
